# American Medical Association

**Published:** 1877-07

**Authors:** 


### American Medical Association.

The recent meeting of the American Medical association, we understand, was of unusual interest—distinguished for concert of feeling and action. We shall be able to furnish complete report in our next number; meanwhile we take great pleasure in announcing that Buffalo is appointed as the place of meeting for the American Medical Association in 1878, by invitation of physicians of Buffalo, and of Erie County.

